# The relationship between lipids level and cerebral microbleeds in patients with spontaneous intracerebral hemorrhage

**DOI:** 10.3389/fneur.2025.1619440

**Published:** 2025-07-18

**Authors:** Hao Feng, Xin Wang, Wenjuan Wang, Xingquan Zhao

**Affiliations:** ^1^Department of Neurology, Beijing Tiantan Hospital, Capital Medical University, Beijing, China; ^2^China National Clinical Research Center for Neurological Diseases, Beijing, China; ^3^Center of Stroke Beijing Institute for Brain Disorders, Beijing, China; ^4^Research Unit of Artificial Intelligence in Cerebrovascular Disease, Chinese Academy of Medical Sciences, Beijing, China

**Keywords:** spontaneous, intracerebral hemorrhage, total cholesterol, non-high-density lipoprotein, cerebral microbleeds

## Abstract

**Background:**

Previous studies indicate a potential link between elevated serum lipid levels and an increased risk of cerebral microbleeds (CMBs). Since multiple CMBs can elevate the risk of future intracerebral hemorrhage (ICH), it is important to explore the relationship between lipid profiles and CMBs in ICH patients. However, data on this specific correlation in ICH populations are currently limited.

**Methods:**

This study retrospectively enrolled 223 consecutive patients from a spontaneous ICH cohort. We collected comprehensive clinical, demographic, and laboratory data, with a focus on lipid levels and the presence of CMBs. Using a multivariate logistic regression model, we investigated the association between lipid parameters and CMB occurrence.

**Results:**

Among the 223 patients, 111 (49.8%) had CMBs. Univariate analysis showed that individuals without lobar CMBs tended to have higher levels of serum total cholesterol (TC), low-density lipoprotein (LDL), and non-high-density lipoprotein (Non-HDL). After adjusting for potential confounders, TC [odds ratio (OR), 0.989; 95% confidence interval (CI), 0.979–0.999; *p* = 0.028] and Non-HDL (OR, 0.989; 95% CI, 0.979–1.000; *p* = 0.043) were identified as independent predictors of lobar CMBs.

**Conclusion:**

Our findings suggest an inverse correlation between TC and Non-HDL levels and the presence of lobar CMBs in ICH patients. Further prospective studies are needed to clarify the causal relationship between statin use and CMBs in ICH patients and to evaluate the prognostic significance of CMB presence and severity in statin-treated individuals.

## 1 Introduction

It is well established that serum cholesterol plays a significant role in the development of atherosclerotic cardiovascular disease. Current guidelines recommend using the maximum tolerated doses of lipid-lowering agents for high-risk patients to reduce low-density lipoprotein cholesterol (LDL) levels. However, there is ongoing debate in clinical practice regarding whether lower cholesterol levels might increase the risk of intracerebral hemorrhage (ICH). A cross-sectional study involving 39,595 patients demonstrated that low LDL levels could potentially increase the risk of ICH (1). Therefore, determining the optimal LDL target requires balancing the risks of ischemia and bleeding.

Cerebral microbleeds (CMBs) are defined as small areas of extremely low signal intensity with a diameter of 2–10 millimeters on T2* gradient echo magnetic resonance imaging (MRI) scans (2). They are considered an imaging marker of vascular fragility. An increase in CMBs is regarded as an imaging marker for identifying individuals at high risk of subsequent ICH. Thus, studying the factors associated with CMBs may help prevent future ICH events. Several studies have reported that decreased serum lipid levels are an independent risk factor for CMBs (3). This suggests that there may be therapeutic implications in exploring the correlation between blood lipid levels and CMBs, particularly in identifying modifiable risk factors for CMBs in ICH patients.

The aim of this retrospective, observational study is to investigate the relationship between lipid levels and the presence of CMBs in patients with spontaneous ICH.

## 2 Subjects and methods

This research protocol has received approval from the Institutional Review Board of Beijing Tiantan Hospital, Capital Medical University (KY2014-023-02). The ethics committee permitted proxy consent as stated in the ethical declaration. All patients participating in the study provided written informed consent.

### 2.1 Study subjects

We conducted a retrospective cohort study using data from patients with ICH admitted to our hospital between January 2014 and August 2016. The data were prospectively and routinely collected. The inclusion criteria were: (1) adult patients aged 18 years or older; (2) patients diagnosed with ICH based on brain computed tomography; and (3) patients for whom information on CMBs, clinical factors, and lipid levels was available. Patients were excluded if they had: (1) primary intraventricular hemorrhage; (2) secondary intracerebral hemorrhage, including cases due to head trauma, brain tumors, aneurysms, cavernous malformations, arteriovenous malformations, acute thrombolysis, coagulopathy, or Moyamoya disease; or (3) undergone surgical intervention before magnetic resonance imaging, such as external ventricular drainage, craniotomy, or hematoma aspiration.

### 2.2 Clinical baseline information

Patient information was gathered from electronic medical records and included the following details: age, gender, smoking habits, alcohol consumption, body mass index (BMI), past medical history (including hypertension, diabetes mellitus (DM), hyperlipidemia, ischemic stroke, ICH, subarachnoid hemorrhage (SAH), use of antiplatelet drugs, anticoagulant drugs, and statins), Glasgow Coma Scale (GCS) score, National Institutes of Health Stroke Scale (NIHSS) score, hematoma location, and primary hematoma volume. Routine laboratory testing was performed with in 1 h of arrival. Blood samples were collected from an antecubital vein the next morning following an overnight fasting (>8 h). White blood cell (WBC) count, platelet count, fasting blood glucose level, international normalized ratio (INR), total cholesterol (TC), triglycerides (TG), high-density lipoprotein (HDL), low-density lipoprotein (LDL), non-high-density lipoprotein (Non-HDL) were recorded.

### 2.3 Imaging analysis

Two trained neurologists, blinded to the clinical data, independently assessed the magnetic resonance imaging (MRI) scans to identify the presence, location, and count of CMBs. MRIs were conducted within 6 days of admission. CMBs were categorized into three types: deep, lobar, and infratentorial, in line with established classification criteria (4). Specifically, lobar CMBs involved the cortex, subcortical structures, and white matter of the frontal, parietal, temporal, occipital, and insular lobes. Deep CMBs included those in the head of the caudate nucleus, putamen, globus pallidus, internal capsule, and thalamus. Infratentorial CMBs encompassed those in the midbrain, pons, medulla oblongata, and cerebellum. The number of CMBs in each location was evaluated on susceptibility-weighted imaging (SWI) and aggregated to determine the total CMB count. To ensure accuracy, microbleeds were distinguished from other findings using CT scans or T2W and FLAIR imaging. Particular care was taken to exclude basal ganglia calcifications, which can resemble microbleeds, by referring to CT scans. Additionally, artifacts mimicking sulcal vessels in the transverse plane and partial volume effects from adjacent bony structures were carefully excluded by examining adjacent slices and consulting available T2W and FLAIR images. The inter-observer agreement for microbleed identification was robust, with a Cohen’s kappa value ranging from 0.96 to 0.98. Discrepancies were resolved by consensus.

### 2.4 Statistical analysis

Continuous variables were expressed as mean (standard deviation) or median (interquartile range) based on the data distribution, while categorical variables were presented as counts and frequencies. Student’s *t*-test or the Mann–Whitney U test was used to compare continuous variables, as appropriate. The chi-square test was employed for comparing categorical variables. Multivariate analysis was performed using a logistic regression model with the enter method to identify independent predictors. In the univariate analysis, variables with a two-sided *p*-value of less than 0.05 were included in the binary logistic regression model. TC, LDL, and Non-HDL were each forced into the model. The odds ratio (OR) and 95% confidence interval (CI) were calculated for each model. All analyses were conducted without access to participants’ identifying information. A *p*-value of less than 0.05 was considered statistically significant. All statistical analyses were carried out using the SPSS software package.

## 3 Results

From our database, we identified 223 participants who had records of both CMBs and serum lipid levels. All the enrolled patients were diagnosed with primary ICH, among them, 192 cases (86.1%) were hypertensive ICH, 9 cases (4.0%) were probable or possible cerebral amyloid angiopathy (CAA), and 22 cases (9.9%) (9.9%) had unknown causes. The average age of the participants was 55 ± 13 years, with an age range of 18 to 86 years, and 163 (73.1%) were male. A total of 206 patients (92.4%) experienced supratentorial ICH, among which 69 cases were lobar hemorrhage, 99 cases were basal ganglia hemorrhage, and 38 cases were thalamic hemorrhage. We observed 637 CMBs in 111 individuals (49.8%). Specifically, 41 (18.4%) patients had lobar CMBs, 90 (40.4%) had deep CMBs, and 42 (18.8%) had infratentorial CMBs. Overall, 8 (3.6%) had only lobar CMBs, 44 (19.7%) had only deep CMBs, and 12 (5.4%) had only infratentorial CMBs.

**Figure 1:**
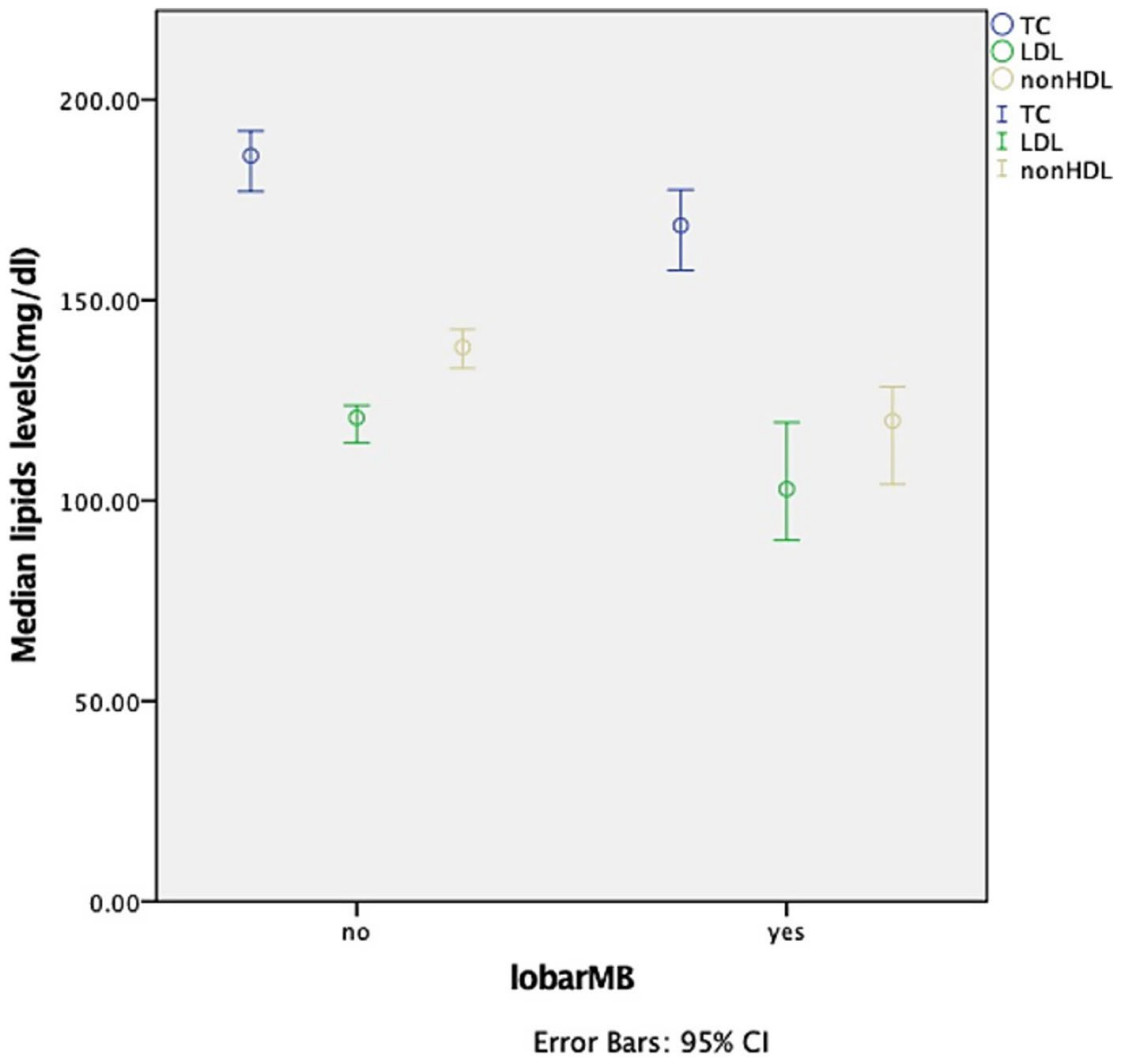
Comparison of lipids levels between the presence and absence of lobar microbleeds.

Tables 1–4 presents the baseline demographics stratified by CMB distribution. A history of hypertension was associated with the presence of any CMBs (*p* = 0.002), lobar CMBs (*p* = 0.037), and deep CMBs (*p* = 0.006). Alcohol intake was linked to the presence of any CMBs (*p* = 0.006), deep CMBs (*p* = 0.038), and infratentorial CMBs (*p* = 0.026). Patients with lobar CMBs tended to be older (*p* = 0.006) and had a higher prevalence of hypertension (*p* = 0.037). Additionally, serum TC levels, LDL levels, and Non-HDL levels were elevated in individuals without lobar CMBs (Figure 1). Following multivariate logistic regression analysis, TC (OR, 0.989; 95% CI, 0.979–0.999; *p* = 0.028) and Non-HDL (OR, 0.989; 95% CI, 0.979–1.000; *p* = 0.043) were independent predictors of lobar CMBs, LDL was not found to be an independent predictor of lobar CMBs after adjusting for age and hypertension history (Table 5).

**Table 1:** Baseline characteristics of patients with and without any microbleed.

	Total *N* = 223	Without any MB *N* = 111	Any MB *N* = 112	*p*
Age (y)	55 ± 13	55 ± 14	56 ± 12	0.548
Male %	163(73.1)	77(69.4)	86(76.8)	0.212
BMI	25.3(22.9–27.6)	25.2(22.9–27.6)	25.4(22.7–27.6)	0.989
History of hypertension %	161(72.2)	70(63.1)	91(81.3)	0.002
History of diabetes	34(15.2)	17(15.3)	17(15.2)	0.977
Smoking				0.723
Never %	111(49.8)	57(51.4)	54(48.2)	
Past %	30(13.5)	16(14.4)	14(12.5)	
Current %	82(36.8)	38(34.2)	44(39.3)	
Alcohol intake %	125(56.1)	52(46.8)	73(65.2)	0.006
Antiplatelet use %	34(15.2)	20(18.0)	14(12.5)	0.252
Anticoagulation %	0	0	0	NA
Statin use %	20(9)	8(7.2)	12(10.7)	0.359
History of CI %	31(13.9)	13(11.7)	18(16.1)	0.347
History of ICH %	5(2.2)	1(0.9)	4(3.5)	0.396
History of SAH %	1(0.4)	1(0.9)	0(0)	0.498
NIHSS	7(3–13)	7(3–13)	8(2–14)	0.750
GCS	15(13–15)	15(14–15)	14(13–15)	0.124
WBC	9.51(7.55–11.88)	9.86(7.88–12.10)	8.97(7.16–11.44)	0.161
Platelet	218 ± 57	218 ± 57	219 ± 58	0.880
Fasting glucose (mmol/L)	5.21(4.47–6.03)	5.05(4.35–6.11)	5.27(4.58–5.97)	0.586
INR	0.95(0.90–1.00)	0.95(0.91–1.01)	0.94(0.89–0.99)	0.157
TC (mg/dL)	181.36(156.61–209.98)	187.16(156.61–216.17)	177.11(156.42–204.18)	0.320
TG (mg/dL)	117.76(85.89–154.07)	118.65(90.32–155.84)	116.44(82.57–150.53)	0.572
HDL (mg/dL)	44.47(38.67–54.91)	44.86(37.90–56.84)	44.47(39.06–54.04)	0.785
LDL (mg/dL)	118.72(93.97–140.76)	121.04(93.19–145.01)	118.33(93.97–138.73)	0.526
Non-HDL	134.96(108.28–159.71)	137.28(109.82–161.64)	132.25(105.28–158.16)	0.402
Volume	13.3(6.0–32.7)	13.1(7.2–28.1)	13.7(5.3–35.1)	0.870
Location				0.816
Supratentorial %	206(92.4)	103(92.8)	103(92.0)	
Infratentorial %	17(7.6)	8(7.2)	9(8.0)	

**Table 2:** Baseline characteristics of patients with and without and lobar microbleed.

	Without lobar MB *N* = 182	Lobar MB *N* = 41	*p*
Age (y)	55 ± 13	61 ± 11	0.006
Male %	134(73.6)	29(70.7)	0.706
BMI	25.4(22.9–27.7)	24.5(22.8–26.4)	0.181
History of hypertension %	126(69.2)	35(85.4)	0.037
History of diabetes	27(14.8)	7(17.1)	0.719
Smoking			0.666
Never %	88(48.4)	23(56.1)	
Past %	25(13.7)	5(12.2)	
Current %	69(37.9)	13(31.7)	
Alcohol intake %	100(54.9)	25(61.0)	0.482
Antiplatelet use %	28(15.4)	6(14.6)	0.904
Anticoagulation %	0	0	NA
Statin use %	16(8.8)	4(9.8)	1.000
History of CI %	23(12.6)	8(19.5)	0.250
History of ICH %	4(2.2)	1(2.4)	1.000
History of SAH %	1(0.5)	0(0)	1.000
NIHSS	8(3–13)	5(2–14)	0.374
GCS	15(13–15)	15(13–15)	0.758
WBC	9.79(7.60–11.89)	8.63(6.40–11.52)	0.133
Platelet	221 ± 58	206 ± 55	0.134
Fasting glucose (mmol/L)	5.19(4.52–5.99)	5.41(4.35–6.42)	0.703
INR	0.95(0.91–1.00)	0.96(0.89–1.00)	0.810
TC (mg/dL)	186.01(158.06–215.01)	168.60(149.85–192.77)	0.012
TG (mg/dL)	120.42(90.32–158.28)	110.68(71.28–139.02)	0.105
HDL (mg/dL)	44.67(37.90–55.01)	44.47(40.41–53.56)	0.841
LDL (mg/dL)	120.65(95.61–143.66)	102.86(85.46–129.93)	0.026
Non-HDL	138.25(109.82–161.83)	119.88(102.29–141.54)	0.006
Volume	13.5(7.2–28.9)	12.5(4.2–38.2)	0.716
Location			0.684
Supratentorial %	167(91.8)	39(95.1)	
Infratentorial %	15(8.2)	2(4.9)	

**Table 3:** Baseline characteristics of patients with and without and deep microbleed.

	Without deep MB *N* = 133	Deep MB *N* = 90	*p*
Age (y)	55 ± 14	57 ± 11	0.368
Male %	97(72.9)	66(73.3)	0.947
BMI	25.4(23.1–27.6)	24.8(22.5–27.6)	0.470
History of hypertension %	87(65.4)	74(82.2)	0.006
History of diabetes	23(17.3)	11(12.2)	0.301
Smoking			0.587
Never %	67(50.4)	44(48.9)	
Past %	20(15.0)	10(11.1)	
Current %	46(34.6)	36(40.0)	
Alcohol intake %	67(50.4)	58(64.4)	0.038
Antiplatelet use %	23(17.3)	11(12.2)	0.301
Anticoagulation %	0	0	NA
Statin use %	11(8.3)	9(10.0)	0.657
History of CI %	16(12.0)	15(16.7)	0.326
History of ICH %	3(2.2)	2(2.2)	1.000
History of SAH %	1(0.8)	0(0)	1.000
NIHSS	7(3–13)	8(2–13)	0.922
GCS	15(13–15)	14(13–15)	0.197
WBC	9.24(7.65–1.95)	9.76(6.99–1.70)	0.730
Platelet	218 ± 57	220 ± 57	0.773
Fasting glucose (mmol/L)	5.31(4.46–6.18)	5.19(4.51–5.91)	0.524
INR	0.96(0.91–1.03)	0.94(0.89–0.97)	0.025
TC (mg/dL)	183.30(155.84–211.91)	177.50(157.97–205.34)	0.675
TG (mg/dL)	120.42(89.88–156.28)	113.34(80.80–148.54)	0.370
HDL (mg/dL)	44.86(37.90–55.69)	44.47(39.44–54.14)	0.800
LDL (mg/dL)	119.49(93.00–141.92)	116.79(93.97–139.70)	0.666
Non-HDL	137.28(108.47–158.94)	132.06(106.73–159.90)	0.559
Volume	13.5(7.4–29.9)	13.0(4.8–35.1)	0.759
Location			0.558
Supratentorial %	124(93.2)	82(91.1)	
Infratentorial %	9(6.8)	8(8.9)	

**Table 4:** Baseline characteristics of patients with and without and infra microbleed.

	Without infra MB *N* = 181	Infra MB *N* = 42	*p*
Age (y)	56 ± 13	55 ± 13	0.548
Male %	130(71.8)	33(78.6)	0.374
BMI	25.4(22.9–27.6)	24.5(22.7–27.8)	0.889
History of hypertension %	128(70.7)	33(78.6)	0.306
History of diabetes	29(16.0)	5(11.9)	0.504
Smoking			0.728
Never %	92(50.8)	19(45.2)	
Past %	23(12.7)	7(16.7)	
Current %	66(36.5)	16(38.1)	
Alcohol intake %	95(52.5)	30(71.4)	0.026
Antiplatelet use %	27(14.9)	7(16.7)	0.776
Anticoagulation %	0	0	NA
Statin use %	16(8.8)	4(9.5)	1.000
History of CI %	24(13.3)	7(16.7)	0.565
History of ICH %	3(1.7)	2(4.8)	0.238
History of SAH %	1(0.6)	0(0)	1.000
NIHSS	8(3–14)	6(2–13)	0.436
GCS	15(13–15)	14(13–15)	0.183
WBC	9.51(7.53–11.84)	9.49(7.58–12.01)	0.815
Platelet	215 ± 57	234 ± 55	0.046
Fasting glucose (mmol/L)	5.21(4.53–6.17)	5.06(4.37–5.90)	0.362
INR	0.95(0.90–1.00)	0.96(0.89–1.00)	0.924
TC (mg/dL)	182.14(157.58–210.36)	173.05(153.43–202.44)	0.295
TG (mg/dL)	118.65(85.45–153.63)	114.67(83.67–160.27)	0.939
HDL (mg/dL)	45.24(39.06–55.69)	42.35(35.48–50.85)	0.092
LDL (mg/dL)	119.49(95.90–140.38)	112.34(85.85–141.24)	0.277
Non-HDL	136.12(109.24–160.10)	132.45(103.93–158.55)	0.503
Volume	14.0(6.9–35.3)	10.7(3.8–26.2)	0.148
Location			0.651
Supratentorial %	166(91.7)	40(95.2)	
Infratentorial %	15(8.3)	2(4.8)	

**Table 5:** Multivariate-adjusted OR and 95% CI of lipids levels for lobar microbleed.

	OR	95%CI	*p*
TC*	0.989	0.979-0.999	0.028
LDL*	0.991	0.980-1.001	0.089
Non-HDL*	0.989	0.979-1.000	0.043

*Adjusted for age and history of hypertension.

## 4 Discussion

Our study demonstrates a correlation between TC levels, Non-HDL levels, and the presence of lobar CMBs in ICH patients. In contrast, we did not find a relationship between serum lipid levels and the incidence of deep CMBs and infratentorial CMBs.

Previous literature has explored the relationship between serum lipids and CMBs, but studies focusing on the ICH population are scarce, and detailed classification of CMB locations has been lacking. A subgroup analysis of the CIRCLE study indicated that lower LDL levels were associated with a higher development of CMBs, while statin therapy did not increase the risk of new CMBs in individuals without a history of stroke (5). However, a prospective cohort study in patients with atrial fibrillation found no association between LDL levels and CMB progression (6). A meta-analysis observed that TC was significantly inversely correlated with prevalent CMBs in any location, while TG and HDL were significantly inversely associated with prevalent deep CMBs after adjusting for covariates. LDL was negatively associated with incident CMBs after controlling for confounders. No statistical differences were found between statin use and CMBs after adjusting for covariates (7). Diogo et al. conducted a retrospective review of their prospectively collected ICH database from 2008 to 2011 and confirmed that statin treatment was associated with the presence and increased number of CMBs, particularly cortico-subcortical CMBs. However, they did not find an association between statin treatment and the presence or number of other types of CMBs (8). Based on the above research, we speculate that the relationship between serum lipids and CMBs may vary across different populations, and our study provides evidence for this.

We revealed an association between lobar CMBs and TC levels and Non-HDL levels rather than LDL levels. Our research findings suggest that TC levels and Non-HDL levels are negatively correlated with the occurrence of lobar CMBs, a conclusion that is consistent with previous researches. Non-HDL includes very-low-density lipoprotein (VLDL), LDL, medium-density lipoprotein (IDL), and lipoprotein (a) (9). Non-HDL is considered superior to LDL in predicting cardiovascular events and has more significant advantages than other lipid profiles (10, 11). The relationship between low lipid levels and CMBs may be explained by the following mechanisms: Decreased lipid levels can increase the permeability of red blood cell membranes, as cholesterol is a crucial component of cell membranes; low cholesterol levels may lead to degeneration of smooth muscle in small cerebral arteries and endothelial fragility, causing arterial fragility and easy leakage; cholesterol can promote platelet activation and tissue factor expression and is related to vitamin K-dependent coagulation factors and fibrinogen, thereby leading to a pro-hemorrhagic state (5, 12–14). Our results also have some discrepancies with previous studies. First, we did not found LDL was correlated with lobar CMBs in the multivariate analysis. One possible reason for this is the sample size is not large enough, which leads to some bias in demographic characteristics. Compared with the research by Yuqi Zhao et al. (5), the proportion of female subjects in our study is lower (26.9% versus 45.5%) and the age is younger (55 ± 13 versus 62.9 ± 10.2). Previous studies have shown that elevated LDL levels increase the risk of hemorrhagic stroke in women (15), and the relationship between lipid levels and hemorrhagic stroke differs between men and women (16). Furthermore, it has been widely confirmed CMB frequency increased with age (17).

In our study, no correlation was found between previous statin use and CMBs, which contradicts previous findings (8). This discrepancy might be due to the relatively low proportion of patients with prior statin use. Actually, the relationship between the use of statins and the risk of ICH and CMBs remains controversial (18). Although the SPARCL study (19) indicated a significantly increased risk of ICH in patients treated with statins (HR, 1.66; 95% CI, 1.08–2.55), subsequent studies have not reached similar conclusions, with some even contradicting this finding. A large-scale case–control study based on the Swedish population demonstrated that statin use before ICH occurrence was associated with a reduced risk of ICH in patients after adjusting for vascular risk factors and antithrombotic therapy use (OR, 0.68; 95% CI, 0.63–0.74) (20). This protective mechanism is thought to be due to the pleiotropic effects of statins, such as their anti-inflammatory and anti-thrombotic properties, which may help protect cerebral blood vessels (21–23).

This study has limitations, first, this is single-center study with mostly Chinese population and its conclusion may not be representative of all ethnic groups. Second, attributed to its retrospective nature and the relatively small number of patients receiving statin therapy, we were unable to determine the temporal relationship between statin use and CMBs, which is crucial for supporting a causal relationship.

## 5 Conclusion

In summary, we found that TC levels and Non-HDL levels are inversely correlated with the presence of lobar CMBs in ICH patients. Future prospective studies are needed to further explore the causal relationship between statin use and CMBs in ICH patients and to evaluate the prognostic value of the presence and severity of CMBs in patients treated with statins.

## Data Availability

The datasets presented in this article are not readily available because the date that supports the findings of this study are available from the corresponding author upon reasonable request. Requests to access the datasets should be directed to Xingquan Zhao, zxq@vip.163.com.
